# Expression of the Ladybird-like homeobox 2 transcription factor in the developing mouse testis and epididymis

**DOI:** 10.1186/1471-213X-8-22

**Published:** 2008-02-27

**Authors:** Vanessa Moisan, Daniela Bomgardner, Jacques J Tremblay

**Affiliations:** 1 Ontogeny-Reproduction Research Unit, CHUQ Research Centre (CHUL), Québec City, Québec, Canada; 2 Centre for Research in Biology of Reproduction, Department of Obstetrics and Gynecology, Faculty of Medicine, Université Laval, Québec City, Québec, Canada; 3 Department of Cell Biology, University of Virginia Health Science System, Charlottesville, Virginia, USA

## Abstract

**Background:**

Homeoproteins are a class of transcription factors that are well-known regulators of organogenesis and cell differentiation in numerous tissues, including the male reproductive system. Indeed, a handful of homeoproteins have so far been identified in the testis and epididymis where a few were shown to play important developmental roles. Through a degenerate PCR approach aimed at identifying novel homeoproteins expressed in the male reproductive system, we have detected several homeoproteins most of which had never been described before in this tissue. One of these homeoproteins is Ladybird-like homeobox 2 (Lbx2), a homeobox factor mostly known to be expressed in the nervous system.

**Results:**

To better define the expression profile of Lbx2 in the male reproductive system, we have performed *in situ *hybridization throughout testicular and epididymal development and into adulthood. Lbx2 expression was also confirmed by real time RT-PCR in those tissues and in several testicular and epididymal cell lines. In the epididymis, a highly segmented tissue, Lbx2 shows a regionalized expression profile, being more expressed in proximal segments of the caput epididymis than any other segment. In the testis, we found that Lbx2 is constitutively expressed at high levels in Sertoli cells. In interstitial cells, Lbx2 is weakly expressed during fetal and early postnatal life, highly expressed around P32-P36, and absent in adult animals. Finally, Lbx2 can also be detected in a population of germ cells in adults.

**Conclusion:**

Altogether, our data suggest that the homeoprotein Lbx2 might be involved in the regulation of male reproductive system development and cell differentiation as well as in male epididymal segmentation.

## Background

Homeobox genes encode transcription factors known as homeoproteins that share a highly conserved 60 amino acid DNA-binding motif called a homeodomain [1-3]. Homeoproteins are known to regulate expression of genes involved in critical developmental and physiological processes in all living organisms. These processes include body plan segmentation, organogenesis, molecular gradient specification, and cell lineage specification and differentiation. Homeoproteins have been identified in several tissues and the male reproductive system is no exception (reviewed in [4,5]).

The male reproductive system is essential for the production of fully functional gametes and for the establishment of the secondary sexual characteristics. It is composed of the testis and several secondary sex organs: the rete testis, epididymis, vas deferens, seminal vesicles, prostate and bulbourethral glands. Proper development of the male reproductive system is thus indispensable for normal male sex differentiation and reproductive function. The process of male sex determination/differentiation is triggered by the Y chromosome-linked *SRY *(Sex-determining Region Y) gene (reviewed in [6]). In the mouse, *Sry *is transiently expressed (between embryonic day 10.5 and E12.5) specifically in pre-Sertoli cells. Since SRY expression is limited to a discrete period of testis differentiation [7,8], it acts as a molecular switch to turn on a network of molecular and cellular events essentials for testicular development as well as male sex differentiation. Three critical hormones produced by the somatic cells of the newly formed testis are essential for male sex differentiation and reproductive function: Müllerian inhibiting substance/anti-Müllerian hormone (MIS/AMH), insulin-like 3 (INSL3), and testosterone (reviewed in [6]). MIS, a hormone belonging to the TGFβ family, is produced by Sertoli cells and regulates male sex differentiation by triggering regression of the Müllerian ducts, which if left intact would develop into the internal female reproductive tract (fallopian tubes, uterus, and upper part of the vagina) [9]. Testosterone, a steroid hormone, secreted by Leydig cells and its more potent derivative dihydrotestosterone regulate several key processes that include testicular descent, development of the accessory sex glands and external genitalia, masculinization of the brain, male sexual behavior, and initiation and maintenance of spermatogenesis (male gamete production) [10]. INSL3, a small peptide belonging to the insulin/relaxin/growth factor family also produced by Leydig cells, regulates the first phase of testis descent during fetal life [11,12] and acts as a germ cell survival factor in adults [13].

Although the testis is the site of spermatogenesis, spermatozoa that exit the testis do not have the capacity to fertilize eggs. The final steps of spermatozoa maturation (acquisition of motility, chromatin condensation) occur in the epididymis, a convoluted and androgen-regulated organ composed of one long tubule divided into three distinct regions called caput, corpus and cauda. Epididymal segmentation is directly related to its function which is species-conserved [14,15]. In addition to functionality, each region of the epididymal tubule is characterized by a distinct physiology. Therefore several genes have been shown to have a region-specific expression along the epididymis tubule [16]. In addition to its importance for sperm maturation, the epididymis also serves as a reservoir for spermatozoa [17].

The process of testis and epididymis formation, as for organogenesis of all tissues, relies on a network of hormones and signaling molecules that act by regulating expression of genes involved in specifying the unique features and functions of these tissues. Some of these genes encode transcription factors. In recent years, some homeoproteins have been implicated in testicular and epididymal development and include Emx2 [18], Lhx9 [19], Pbx1 [20], Arx [21], HoxA10 [22], and Pax2 [23].

Here we report the identification, through a degenerate PCR approach, of additional homeobox factors expressed in the male reproductive system. In addition, we have performed a detailed characterization of the expression profile during testicular and epididymal development of one of the homeoprotein identified, the Ladybird-like homeobox 2 (Lbx2) homeoprotein.

## Results and Discussion

**Figure 1:**
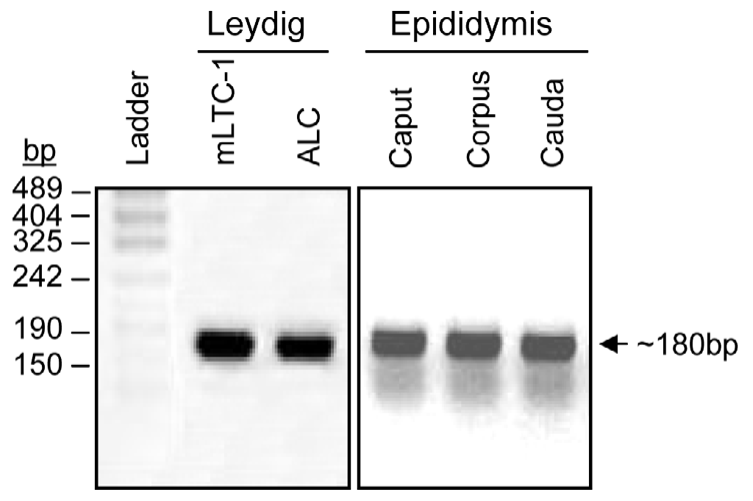
**Identification of homeobox factors in testicular Leydig cells and in mouse epididymis**. A degenerate PCR was performed using cDNAs from mLTC-1 Leydig cell line, purified Leydig cells from adult rats (ALC) and from all three segments of the mouse epididymis and resulted in the amplification of a 180 bp fragment. The fragments were subcloned in pBluescript and sequenced to determine the nature of the homeoprotein (see Table 1).

**Figure 2:**
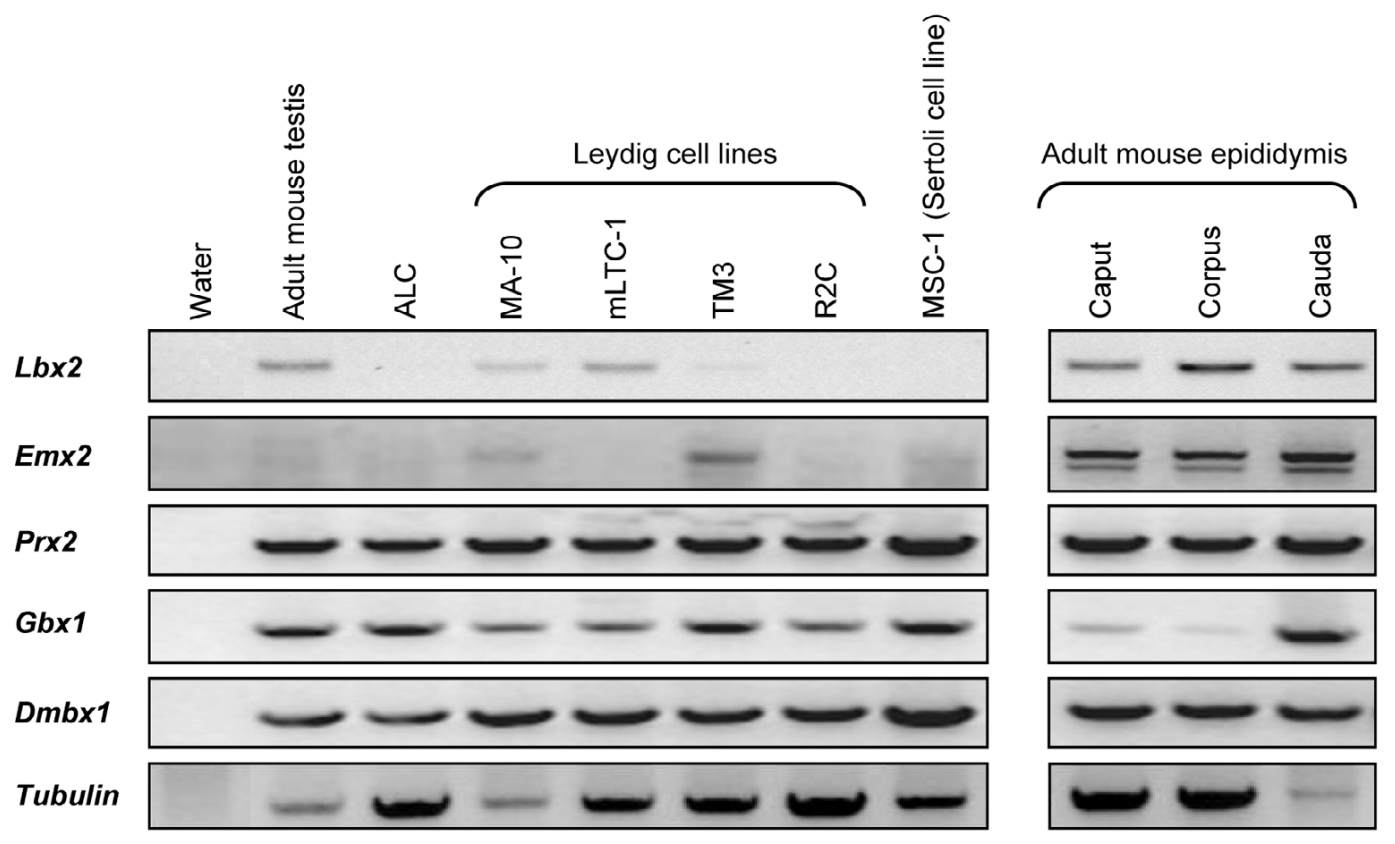
**Expression of five homeoproteins in the testis and epididymis**. PCR reactions were performed using sequence-specific primers (see Table 2) for each of the indicated homeoproteins along with cDNAs from adult mouse testis, purified Leydig cells from adult rats (ALC), various Leydig cell lines (MA-10, mLTC-1, TM3, R2C), a Sertoli cell line (MSC-1), and the three regions of adult mouse epididymis (caput, corpus, cauda). The integrity and amount of cDNA used in the PCR assays was assessed by amplifying tubulin mRNA. No template: negative control.

To identify novel homeoproteins in the male reproductive system, we used a degenerate PCR strategy (see Additional file 1: Degenerate PCR strategy). A mixture of degenerate primers designed to amplify the homeodomain region was used along with cDNAs from mouse Leydig cells and epididymis as template. As shown in Fig. 1, a 180 bp fragment corresponding to the homeodomain was successfully amplified. Because degenerate primers were used, this band is likely composed of various homeodomain sequences. To determine the nature of these homeodomains, the PCR products were subcloned and several independent clones were sequenced. As shown in Table 1, we found nearly a dozen different homeoproteins in Leydig cells and six in the epididymis. Some of these homeoproteins belong to the Hox family but the majorities are non-Hox homeoproteins (Table 1). Interestingly, none of the non-Hox homeoproteins had previously been reported in Leydig cells and in the epididymis, although some (Lbx2, Dmbx1, Emx2, Pbx1) have been detected in the urogenital ridge/testis [18,20,24-26]. To confirm that some of the non-Hox homeoproteins identified in our screen are indeed expressed in male reproductive organs, RT-PCRs with sequence-specific primers (Table 2) were performed using cDNA from testicular tissues and cell lines (Leydig and Sertoli) as well as from the three regions of the epididymis (Fig. 2). As shown in the left panel of Fig. 2, Prx2, Dmbx1, and Gbx1 were detected in all testicular cell lines/tissues tested while Lbx2 and Emx2 were only detected in some testicular cell lines (Fig. 2, left panel). Lbx2 was present in adult mouse testis as well in the Leydig cell lines MA-10 and mLTC-1 whereas Emx2 was detected in MA-10 and TM3 Leydig cell lines. In the epididymis, all homeoproteins analyzed by this technique were detected (Fig. 2, right panel). Our results are consistent with previous data reporting expression of Emx2 and Dmbx1 in the urogenital system and testis [18,25,27,28]. Since Lbx2 was detected in both tissues and since scarce information was available regarding its expression in the reproductive system, this homeobox factor was chosen for further analyses.

**Table 1:** Homeoproteins identified by degenerate PCR in Leydig cells and epididymis

**Leydig**	**Epididymis**
Dmbx1	Hoxb-2, Hoxb-3
Emx2	Lbx2
Gbx1, Gbx2	Mox1
Hoxb-2, Hoxb-3, Hoxb-9	Msx1
Hoxc-13	Prx2
Lbx2	
Prx2	
Sax2	

Lbx2 is the second member of a family that also comprises Lbx1 and Lbx3. The mammalian Lbx1 and Lbx2 gene are the homologs of the Drosophila *Ladybird *genes *Ladybird late *(*Lbl*) and *Ladybird early *(*Lbe*). *Ladybird-like *genes were also identified in the chick embryo, Lbx1 and Lbx3, which share a high degree of homology with mammalian *Lbx *genes [29]. In Drosophila, *Lbl *and *Lbe *have been shown to play important roles in neurogenesis, myogenesis, and cardiogenesis [30-33]. A consensus DNA binding site for Lbl and Lbe proteins, RVYTAAYHAG, was recently identified [34]. This motif was then used in a ChIP-enriched *in silico *target approach (ChEST) that led to the identification of several target genes regulated by the Ladybird factors in Drosophila [34]. These genes were found to encode proteins involved in cardiac and muscle cell fate specification as well as in cell shape, adhesion, and motility [34]. Interestingly, in mammals *Lbx1 *was reported to play equally important roles. Indeed, *Lbx1*^-/- ^mice have important defects in heart looping [35], interneuron specification in the spinal cord [36-38], and migration of muscle cell precursors [39-41]. As for Lbx3, its role in chick remains unknown and no mammalian homolog has been identified yet.

**Figure 3:**
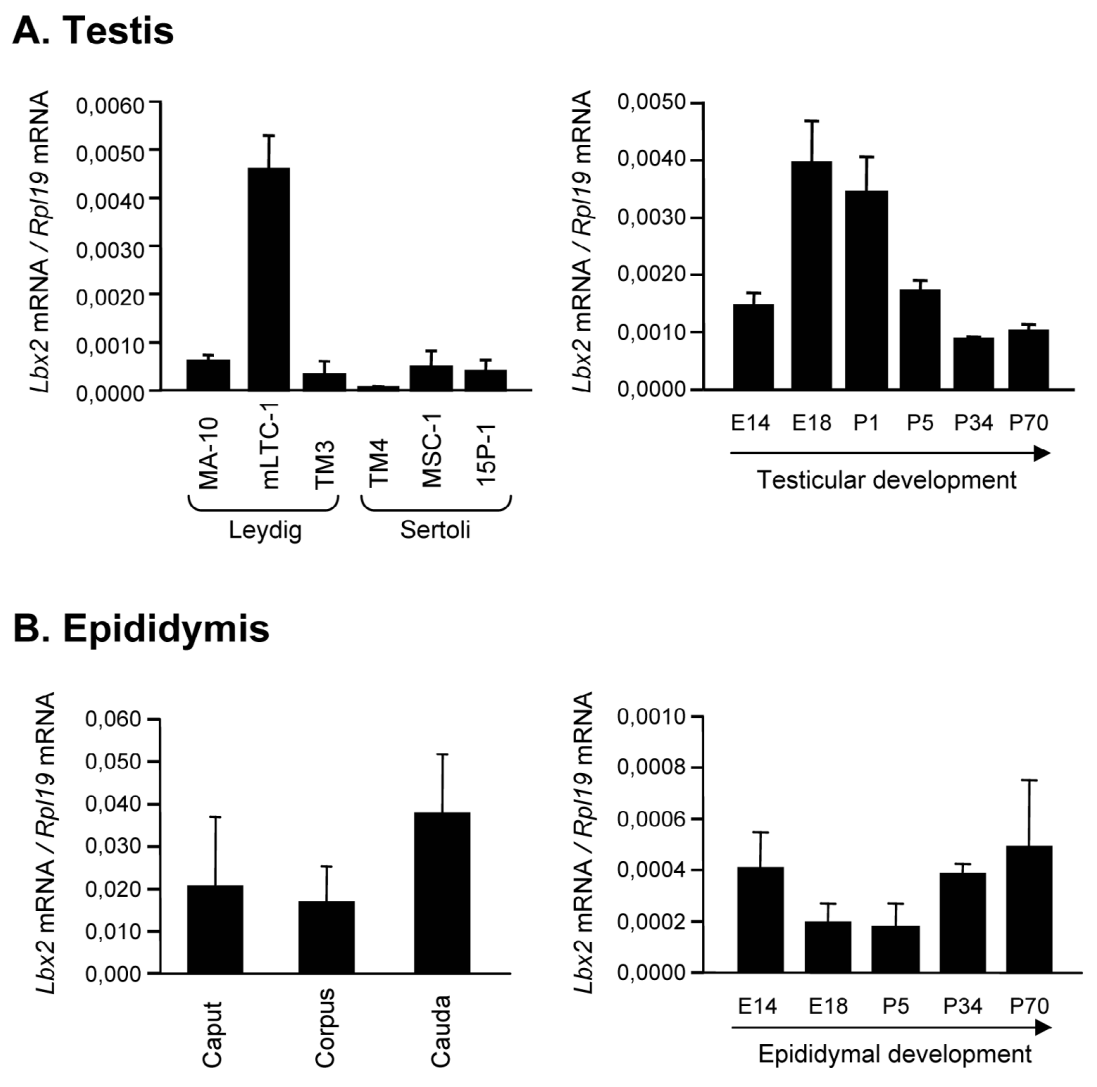
**Lbx2 is expressed in the testis (A) and epididymis (B)**. Quantitative real time PCR were performed with primers specific for Lbx2 cDNA as described in Methods using first strand cDNAs from Leydig cell lines (MA-10, mLTC-1, TM3), Sertoli cell lines (TM4, MSC-1, 15P-1), mouse testis at various developmental ages (E14, E18, P1, P5, P34 and P70), epididymal regions (caput, corpus, cauda) from adult mice, and mouse epididymis at different developmental stages as indicated. Results were corrected with the Rpl19 cDNA. Results are the mean of three individual experiments each performed in duplicate (± SEM).

To confirm Lbx2 expression in the testis and epididymis, we initially performed real time RT-PCR using primers specific for Lbx2 on first strand cDNAs from various sources. We used cDNAs from a panel of cell lines corresponding to Leydig and Sertoli cells and found that Lbx2 was expressed at similar levels in all cell lines tested, except for mLTC-1 Leydig cells which express higher levels of Lbx2 (Fig. 3A, left panel). Similar results for the Leydig cell lines were obtained by Northern blot (Additional file 2: Lbx2 Northern blot in Leydig cell lines). To gain insights into the developmental expression profile of Lbx2 in the testis, real time PCR was performed on cDNAs isolated from testes at various embryonic and postnatal developmental stages. As shown in the right panel of Fig. 3A, we found that Lbx2 was present at all stages tested. Lbx2 mRNA was detected at embryonic day 14 (E14), peaked by E18 and postnatal day 1 (P1), and subsequently decreased by P5 to a level that remained stable throughout adult life (P34 and P70). A similar real time PCR approach was used to assess Lbx2 expression in the epididymis. As shown in the left panel of Fig. 3B, Lbx2 mRNA was expressed at similar levels in the three regions of the adult mouse epididymis (caput, corpus, and cauda). Lbx2 was also expressed at a constant level throughout embryonic and postnatal epididymal development (Fig. 3B, right panel).

**Figure 4:**
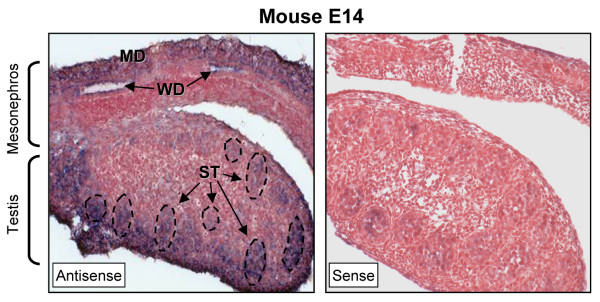
**Lbx2 is strongly expressed in mesonephric cells and in seminiferous tubules of the developing testis**. Six μm paraffin sections of paraformaldehyde-fixed mouse developing urogenital ridge (E14) were probed with DIG-labeled antisense (left panel) and sense (right panel) Lbx2 cRNA probes in *in situ *hybridization experiments. Lbx2 mRNA was detected by immunostaining using an alkaline phosphatase-coupled anti-DIG antibody (appears as a blue-purplish staining). Tissues were counterstained with Neutral Red to visualize nuclei. Lbx2 mRNA was detected in cells of the Wolffian duct (WD), of the degenerating Müllerian duct (MD), and in the developing seminiferous tubules (ST, outlined by dotted lines). Magnification: 200×.

Although the presence of Lbx2 in the testis and epididymis has been confirmed (Figs. 2 and 3), the exact cell type expressing this factor within these tissues remained uncertain. To answer this question, we analyze Lbx2 expression and localization by *in situ *hybridization on tissue sections using a DIG-cRNA probe. Each *in situ *hybridization analysis was performed using three different cRNA probes which all gave similar results. Consistent with the real time PCR data (Fig. 3), Lbx2 is already expressed in the developing male gonad and mesonephros at E14 in the mouse (Fig. 4). In the mesonephros, Lbx2 is present in cells of the Wolffian duct (WD), the anlagen of several male reproductive organs, including the epididymis. Staining is also detected in the lateral part of the mesonephros which would be in agreement with the location of the degenerating Müllerian duct (MD) at that age [42,43]. In the testis, Lbx2 specifically labels cells within the developing seminiferous tubules (ST; dotted lines in Fig. 4) and not the interstitial cells (Fig. 4). Our findings are in agreement with previous data in the literature where Lbx2 was reported to be expressed in several embryonic tissues, including the urogenital ridge between E10.5 and E14.5 in the mouse [24,26]. No information, however, is available regarding Lbx2 expression in reproductive organs at later developmental stages and in adult animals.

**Figure 5:**
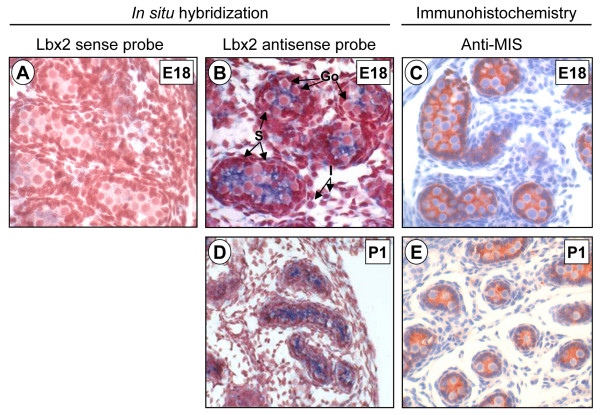
**Lbx2 expression in embryonic an neonatal testis**. Six μm paraffin sections of paraformaldehyde-fixed mouse testis were probed with DIG-labeled antisense (B and D) and sense (A) Lbx2 cRNA probes in *in situ *hybridization experiments. Lbx2 mRNA was detected by immunostaining using an alkaline phosphatase-coupled anti-DIG antibody (appears as a blue-purplish staining). Tissues were counterstained with Neutral Red to visualize nuclei. Lbx2 expression in the testis was assessed at E18 (B) and P1 (D). Immunohistochemistry for the Sertoli cell marker MIS was performed on testis sections at E18 (C) and P1 (E) as described in Methods and revealed using AEC (shows as a red-brownish staining). Expression of Lbx2 is evident within the cytoplasm of Sertoli cells by comparison with MIS staining. A weak but consistent Lbx2 staining is also observed in interstitial cells. Gonocytes are not labeled. No significant signal was detected with the sense probe (A). Go: gonocytes, S: Sertoli cells, I: interstitial cells. Magnifications: 200× (D, E), 400× (A-C).

**Figure 6:**
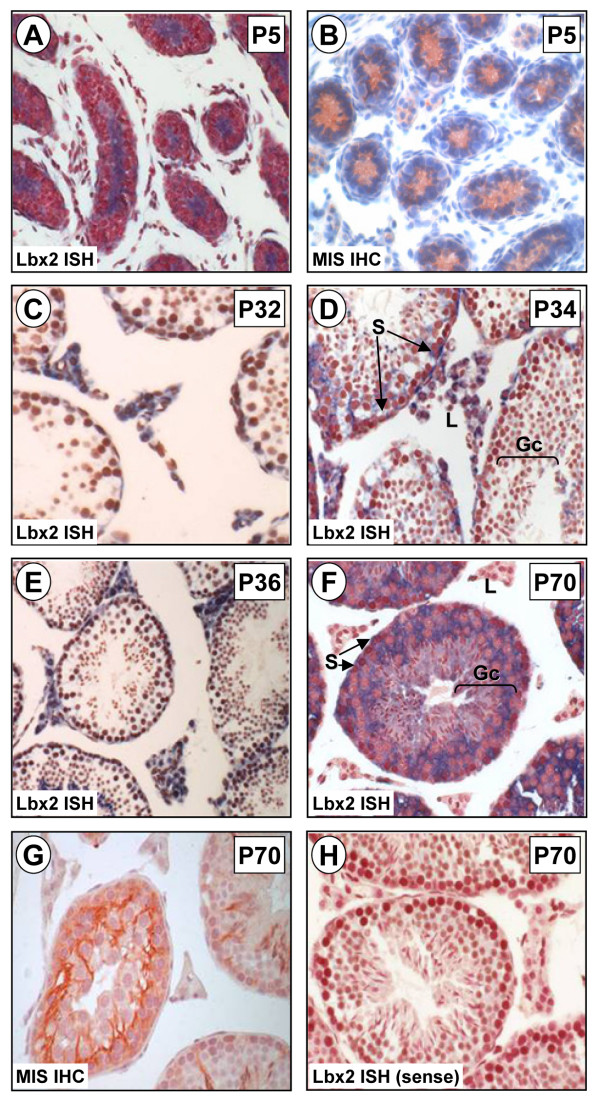
**Lbx2 expression in postnatal testis**. *In situ *hybridization (ISH) experiments using DIG-labeled antisense (A, C, D, E, F) and sense (H) Lbx2 cRNA probes were performed on 6 μm paraffin sections of paraformaldehyde-fixed mouse testis. Lbx2 mRNA was detected by immunostaining using an alkaline phosphatase-coupled anti-DIG antibody (appears as a blue-purplish staining). Tissues were counterstained with Neutral Red to visualize nuclei. Lbx2 expression in the post-natal testis was assessed at P5 (A), P32 (C), P34 (D), P36 (E), and P70 (F). By immunohistochemistry (IHC), the Sertoli cell cytoplasm in P5 (B) and P70 (G) testis was labeled using an anti-MIS antiserum and revealed using AEC (shows as a red-brownish staining). Expression of Lbx2 is evident within the cytoplasm of Sertoli cells at all ages. In interstitial cells, a weak but consistent staining is observed at P5 (A) while a strong signal is detected at P32, P34, and P36 (C-E). At P70, some germ cells are also labeled for Lbx2 (F). No significant signal was detected with a sense probe as shown in the adult (H) section. Gc: germ cells, L: Leydig cells, S: Sertoli cells. Magnifications: 200× (A, B), 400× (C-H).

To address this issue, we performed *in situ *hybridization throughout fetal and postnatal testicular development in the mouse. Lbx2 mRNA was detected in cells within the seminiferous tubules at all ages tested (E18, P1, P5, P32, P34, P36 and P70). In the embryonic (E18) and neonatal (P1) testis, the lumen of the tubules is positive for Lbx2 (Figs. 5B and 5D) which indicates expression in Sertoli cells since at this age their cytoplasm is known to fill the tubule before the onset of spermatogenesis. This was further confirmed by comparing Lbx2 mRNA staining with that of the MIS protein, a well-known marker of Sertoli cells specifically found in the cytoplasm (Fig. 5C and 5E). Gonocytes, located in the tubules, are not labeled for Lbx2 as their small and round cytoplasm can easily be seen as an unlabelled halo around their large nucleus (Fig. 5). Later during postnatal development (P5, P32, P34, P36, P70), Lbx2 expression remained high in cells within the seminiferous tubules that can be identified as Sertoli cells (Figs. 6A, 6C, 6D, 6E, and 6F) by comparing with MIS immunostaining (Figs. 6B and 6G). In addition, some germ cells are also positive for Lbx2 at P70 (Fig. 6F). Since Sertoli cells are the main supporting cells for testicular development and spermatogenesis [44], our data would be consistent with a role for Lbx2 in these processes. In addition to its expression in cells of the seminiferous tubules, Lbx2 could also be detected in interstitial cells. Low staining could be consistently detected in interstitial cells between E18 and P5 (Figs. 5B, 5D, and 6A). This would indicate that Lbx2 is weakly expressed in the fetal Leydig cell population. Fetal Leydig cells are responsible for production of androgens during fetal life and are known to disappear within the first week after birth [45]. Another population of Leydig cells, called the adult Leydig cell population, begins to differentiate around the second week after birth and is responsible for the production of androgens throughout postnatal life [46,47]. Contrary to the fetal Leydig cells, we found that Lbx2 is highly expressed in the adult Leydig cell population around P32, P34 and P36 (Figs. 6C, 6D, and 6E) whereas in mature animals (P70), Leydig cells no longer expressed Lbx2 (Fig. 6F). Taken together our data indicate that Lbx2 is expressed at low levels in fetal Leydig cells but at high levels during the differentiation of the adult population of Leydig cells. This raises the possibility that Lbx2 could regulate specific steps of the Leydig cell differentiation process.

**Figure 7:**
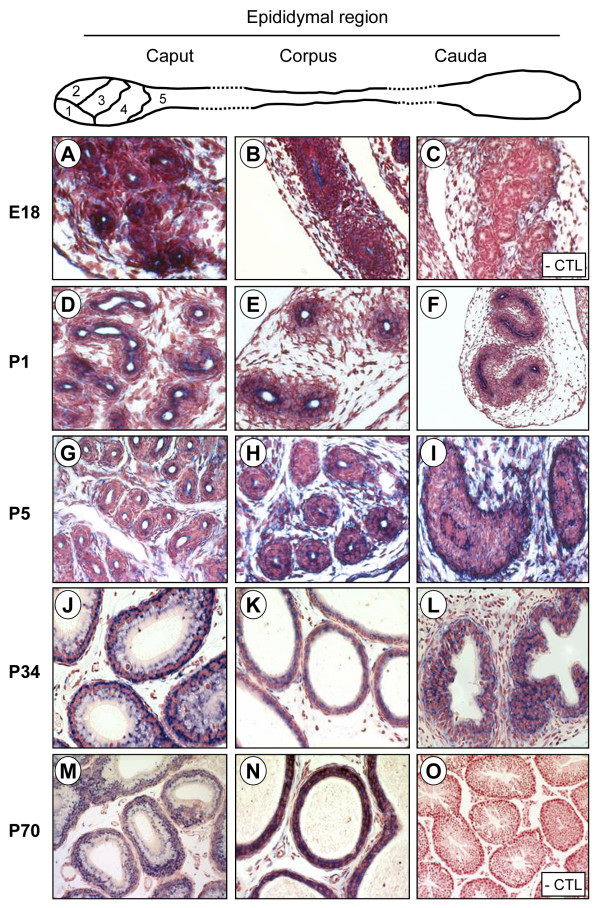
**Lbx2 is strongly expressed throughout epididymal development**. Top panel: schematic representation of the three regions of the epididymis and of the 5 segments of the caput. *In situ *hybridization experiments were performed on six μm paraffin sections of paraformaldehyde-fixed mouse epididymis using DIG-labeled antisense (A, B, D-N) and sense (C, O) Lbx2 cRNA probes. Lbx2 mRNA was detected by immunostaining using an alkaline phosphatase-coupled anti-DIG antibody (appears as a blue-purplish staining). Tissues were counterstained with Neutral Red to visualize nuclei. Lbx2 expression was assessed at different developmental stages, E18 (A, B), P1 (D-F), P5 (G-I), P34 (J-L), P70 (M, N), and in the three regions, caput (A, D, G, J, M), corpus (B, E, H, K, N), cauda (F, I, L) of the epididymis. No significant signal was detected with a sense probe as shown in the E18 (C) and adult (O) sections (- CTL). Magnifications: 100× (C, F, O); 200× (A, B, D, E, G, H, K, M); 400× (I, J, L, N).

**Figure 8:**
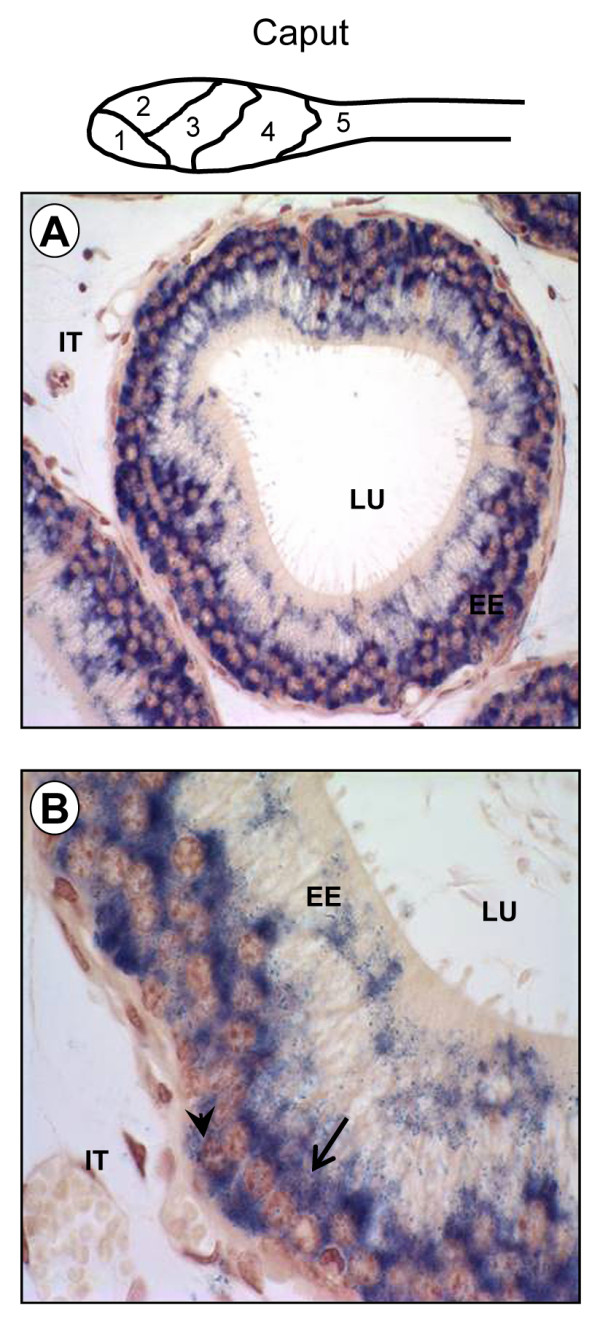
**Lbx2 is expressed in the principal and basal cells of the epididymal epithelium**. Top panel: schematic representation of the caput epididymis and its 5 segments. A DIG-labeled antisense Lbx2 cRNA probe was used in *in situ *hybridization experiments on six μm paraffin sections of paraformaldehyde-fixed mouse adult epididymis. Lbx2 mRNA was detected by immunostaining using an alkaline phosphatase-coupled anti-DIG antibody (appears as a blue-purplish staining). Tissues were counterstained with Neutral Red to visualize nuclei. (A) 400× magnification of the segment 2 of the caput epididymis that reveals strong signal in the epididymal epithelium (EE). (B) The presence of Lbx2 can be observed in principal (arrow) and basal (arrowhead) cells using a 1000× magnification of (A). EE: epididymal epithelium; IT: interstitial compartment; LU; epididymal lumen.

**Figure 9:**
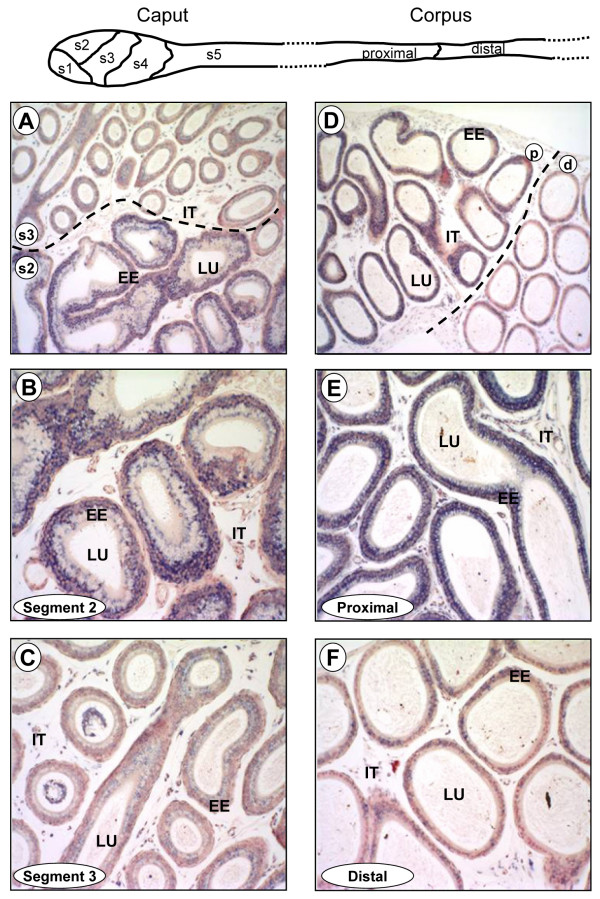
**Lbx2 is expressed in a segment-specific manner in the adult epididymis**. Top panel: schematic representation of the five segments of the caput and the two regions of the corpus epididymis. Six μm paraffin sections of paraformaldehyde-fixed mouse epididymis were probed with a DIG-labeled antisense Lbx2 cRNA probe in *in situ *hybridization experiments. Lbx2 mRNA was detected by immunostaining using an alkaline phosphatase-coupled anti-DIG antibody (appears as a blue-purplish staining). Tissues were counterstained with Neutral Red to visualize nuclei. Caput (A, B, C) and corpus (D, E, F). (A) Localization of Lbx2 in segment 2 (s2) and 3 (s3) of the caput. The two segments are separated by a dotted line. (B) Magnification of segment 2 seen in A. (C) Magnification of segment 3 seen in A. (D) Lbx2 expression in the proximal (p) and distal (d) corpus. A dotted line separates the proximal and distal corpus. (E) Magnification of the proximal corpus seen in D. (F) Magnification of the distal corpus seen in (D). EE: epididymal epithelium; IT: interstitial compartment; LU: epididymal lumen. Magnifications: 100× (A, D); 200× (B, C, E, F).

The detection of Lbx2 expression in cells of the Wolffian duct at E14 (Fig. 4) prompted us to test whether Lbx2 was expressed in the epididymis during mouse development since this tissue derives from the Wolffian duct. The epididymis is a segmented and regionalized organ [16] and homeoproteins, such as the HOX proteins, are essential regulators of body plan segmentation in the antero-posterior axis [48]. As shown in Fig. 7, Lbx2 mRNA was detected in all three regions of the epididymis from E18 to adulthood. At the cellular level, Lbx2 was found to be expressed in principal and basal cells of the epididymal epithelium in adult male mice (Fig. 8). Lbx2 did not label clear and narrow cells (data not shown). Interestingly, we found that Lbx2 shows a highly compartmentalized expression profile in the caput and corpus epididymis (Fig. 9). Indeed, in the caput, which is a highly segmented region of the epididymis [14], Lbx2 was found to be more abundantly expressed in segment 1 (data not shown) and 2 (Figs. 9A and 9B) compared to segment 3 (Figs. 9A and 9C), 4 and 5 (data not shown). Similarly, Lbx2 expression was stronger in the proximal than in the distal corpus (Figs. 9D, 9E, and 9F). So far, only a handful of homeoproteins have been shown to have regionalized expression in the male reproductive tract and most of them belong to the Hox or Meis (Hox-interacting proteins) families [49]. Other transcription factors such as Pea3 and Rhox5 (a member of the reproductive homeobox gene cluster) are also known to be differentially expressed along rodent epididymis [5,50,51]. To this date however, only *Hoxa10 *and *Hoxa11 *have been shown to be involved in segment identity *in vivo *the mouse epididymis [52,53].

Although Lbx2 was found to be strongly expressed in both tissues throughout development into adulthood, we did not detect expression of the highly related Lbx family member Lbx1 (data not shown). This is consistent with the fact that Lbx1 and Lbx2 do not have overlapping expression patterns in general [24,54] and are therefore believed to play non-redundant roles during development. The expression pattern of Lbx2 in the testis and epididymis described herein supports the notion that this transcription factor might be involved in the development and/or the function of these organs. While this manuscript was in preparation, *Lbx2 *null mice have been reported [26]. Surprisingly, *Lbx2*^-/- ^mice are viable and show no gross morphological defects and both male and female *Lbx2*^-/- ^mice were found to be fertile, although no detailed analyses of the reproductive system were reported [26]. A mild partial lethality associated with *Lbx2 *deficient mice was however observed but failed to reach statistical significance [26]. As suggested by Wei *et al*, back crossing the Lbx2 mutation in a different genetic background may be required to detect a phenotype associated with Lbx2 deficiency [26]. Besides the genetic background, this lack of a penetrant phenotype might be explained by a redundancy mechanism where another homeobox factor could compensate for the absence of *Lbx2*. This is very common amongst genes that are essential for development and cell differentiation, including homeobox encoding genes [55-57]. Wei *et al *proposed that the *Tlx2 *homeobox gene is an attracting candidate as a substitute for *Lbx2 *for several reasons. First, Tlx2 and Lbx2 belong to the superclass of homeobox proteins [58]. Second, the genomic location and organization of the *Tlx2 *and *Lbx2 *genes are conserved in Drosophila and mice [58]. And finally, *Tlx2 *and *Lbx2 *expression patterns are overlapping in numerous tissues including the testis [24,26,59]. Besides *Tlx2*, it is also possible that other yet unidentified *Lbx *family members could also compensate for the absence of Lbx2. In agreement with this is the identification of Lbx3 in the avian genome, although a mammalian homolog has yet to be identified [29]. If it does indeed exist, this other *Lbx *family member would represent an interesting candidate to compensate for the absence of *Lbx2*.

## Conclusion

In conclusion, our present study provides new insights into the expression profile of the homeobox factor Lbx2 throughout development of the testis and the epididymis. The lack of overt phenotype in Lbx2 *null *mice may indicate that Lbx2 does not play a dominant role in the development and the function of these organs. Another possibility is that other homeobox factors compensate for the absence of Lbx2. Since Lbx2 expression is dynamic in the testis and epididymis, Lbx2 constitutes a useful molecular marker for histological and developmental studies.

## Methods

### Animals

C57BL/6 mice were maintained on a 12L:12D light cycle with water and food *ad libitum*. Mice were killed at different time points as indicated in the figure legends and the testes and epididymides were harvested. Whole testis and epididymis were fixed in 4% (w/v) paraformaldehyde for 24 h. Tissues were then dehydrate with ethanol, substituted with xylene, and embedded in paraffin. All experiments complied with the regulations set by the Animal Welfare Act (Public Law 91-579), the Canadian Council for Animal Care, the Guide for the Care and Use of Laboratory Animals (National Research Council, 1996) published by the Department of Health and Human Services, and the policies and procedures of the University of Virginia Institutional Animal Care and Use Committee. All experiments have been approved by the Animal Care and Ethics Committee of Laval University (protocol # 06-059).

### Cell culture

Most cell lines used in the present study were obtained from ATCC (Leydig: mTLC-1, TM3, R2C; and Sertoli: TM4, 15P-1). The MA-10 Leydig cell line [60] was provided by Dr. Mario Ascoli (University of Iowa, Iowa City, IA) and the Sertoli MSC-1 cell line was a gift from Dr. Michael Griswold (Washington State University, Pullman, WA). The MSC-1 cell lines were grown in Dulbecco modified Eagle medium (DMEM) supplemented with 10% fetal bovine serum, HEPES and 50 mg/liter of penicillin and streptomycin sulfates. MA-10, were grown in Waymouth's MB752/1 medium supplemented with 20 mM HEPES, 15% horse serum and 50 mg/liter of penicillin and streptomycin sulfates. All cell lines obtained from ATCC were cultured as recommended by ATCC. Cell lines were grown at 37°C and 5% CO_2_.

### RNA preparation and RT-PCR

Total RNA from adult mouse testis, epididymal segment and the various cell lines was isolated using RNeasy Plus extraction kit (Qiagen, Mississauga, Ontario, Canada). First strand cDNAs were synthesized from a 2.5 μg aliquot of the various RNAs using the Transcriptor Reverse Transcriptase kit (Roche Diagnostics, Laval, Canada). The degenerate PCR primers were designed by aligning the sequence encoding the homeodomain of 16 homeoproteins (Additional file 1: Degenerate PCR strategy). The sequences of the degenerate primers are as follow: forward 5'-GAT CTA GAS CAR CTG SAG GMG CTG GAG-3' and reverse 5'-GCG GTA CCG CBC KSC GGT TCT KRA ACC A-3'. The degenerate primers, which are located at each end of the homeodomain, were used in PCR using first strand cDNAs from Leydig cells purified from adult rats (ALC), mLTC-1 Leydig cell line and mouse caput, corpus, cauda epididymis. Total RNA from ALC was kindly provided by Dr. Matthew Hardy, (The Population Council, Rockefeller University, New York, NY). The procedure for cell isolation has been described previously [61] and Leydig cells are typically enriched more than 95% as determined by histochemical staining for 3β-hydroxysteroid dehydrogenase activity [61]. As determined in Dr. Hardy's laboratory, expression of marker genes for other testicular cell types (Sertoli, myoid, lymphocytes and blood cells) was undetectable. The degenerate PCRs were done on a T_gradient _thermocycler (Biometra) using the following conditions: 3 min at 94°C followed by 30 cycles of 1 min at 94°C, 1 min at 51–59°C, 30 sec at 72°C, and a final extension of 5 min at 72°C. The PCR products were subcloned and sequenced. The sequences of the primers specific for Lbx2, Emx2, Prx2, Gbx1, Dmbx1, and tubulin are listed in Table 2. The PCRs were done on a T_gradient _thermocycler (Biometra) using the following conditions: 3 min at 94°C followed by 30 cycles of denaturation (50 sec at 94°C), annealing (1 min at various temperatures; see below), extension (1 min at 72°C), and a final extension of 5 min at 72°C. The annealing temperatures were 58°C for Lbx2, Prx2, tubulin and 60°C for Emx2, Gbx1, and Dmbx1. The PCR products were subcloned in pBluescript (Stratagene) and sequenced on an ABI 3730/XL automated sequencer (Centre de génomique de Québec, Québec City, Canada) to confirm the nature of the amplified cDNAs. The real-time PCRs were carried out using a LightCycler 1.5 instrument from Roche Diagnostics, Laval, Canada. Reactions were performed according to the manufacturer's recommendations. PCRs were performed using the following Lbx2-specific primers: forward, 5'-GAC TGG GCC TGG CTA AT-3' and reverse, 5'-CAG GGT CAG GGC TTG AA-3'. As an internal control, PCRs were performed using previously described Rpl19-specific primers [62]. The PCRs were done using the following conditions: 10 min at 95°C followed by 35 cycles of denaturation (5 sec at 95°C), annealing (5 sec at 62°C for both Rpl19 and Lbx2 cDNAs), and extension (20 sec at 72°C) with single acquisition of fluorescence at the end of each extension steps. After amplification, the samples were slowly heated at 0.2°C/sec from 68°C to 95°C with continuous reading of fluorescence to obtain a melting curve. The specificity of each PCR product was then determined by using the melting-curve analysis program of the LightCycler software. The Lbx2 and Rpl19 PCR products showed a single peak in the analysis. Quantification of gene expression was performed using the Relative Quantification Software (Roche Diagnostics, Laval, Canada) and is expressed as a ratio of Lbx2 to Rpl19 mRNA levels. Each amplification were performed in duplicate using three different preparations of first-strand cDNAs for each of the two different RNA extractions

**Table 2:** Sequence-specific primers used in the RT-PCR studies

**Gene**	**Sequence**
Lbx2	Forward: 5'-ATGGGTACCCGAAGCACCTTCTGCACCGC-3' Reverse: 5'-GCGAATTCAATCGTCCACCTGTATCTCCTC-3
Prx2	Forward: 5'-GCGAATTCAACAGCAGCCAGCTGCAGGCGC-3' Reverse: 5'-GGCCTCGAGGCGAAGGCTGGCGATGCTGTTGGA-3'
Gbx1	Forward: 5'-GCTCTAGAGGGAAGGTGTACAGCTCAGATG-3' Reverse: 5'-CGGGATCCATCTGTTGGTGCTGGCTGCGC-3'
Dmbx1	Forward: 5'-CGGAATTCAATTGGGGAGTGTATCGAGTCCC-3' Reverse: 5'-GAGGATCCCAAAGCTGAAAAGAGCCC-3
Emx2	Forward: 5'-GCTCTAGAGTTCCTCAACGGATTCCACTC-3' Reverse: 5'-GGGGTACCATTTCCTCCGGACTCGCCTGC-3'
Tubulin	Forward: 5'-TCCATCCACGTCGGCCAGGCT-3' Reverse: 5'-GTAGGGCTCAACCACAGCAGT-3'

### *In situ *hybridization

Three different probes for Lbx2 were tested. A 604 bp fragment that encompasses the entire coding sequence (nt 60–624 of Genbank accession number NM_010692), a 504 bp fragment from nt 120 to 624, and a 263 bp fragment that contains the coding sequence C-terminal of the homeodomain into the 3' UTR (nt 490–753). The fragments were obtained by PCR and cloned into pBluescript (Stratagene). Sense and antisense digoxigenin (DIG)-labeled riboprobes for Lbx2 were subsequently obtained by linearizing the plasmid followed by *in vitro *transcription using T7 or T3 RNA polymerase (GE Healthcare) in the presence of DIG-UTP (Roche Diagnostics, Laval, Canada). The DIG-labeled riboprobes were then used in *in situ *hybridization experiments on paraformaldehyde-fixed, paraffin-embedded tissue sections. In brief, testis and epididymis sections were dewaxed in xylene, rehydrated in graded alcohols (95%, 70%, and 50%) and diethylpyrocarbonate-treated water, and digested by proteinase K (10 mg/mL) for 15 min. Glycine (2 mg/mL) was used to stop the proteinase K digestion. Tissues were then refixed with 4% paraformaldehyde and treated with 0.25% acetic anhydride in 0.1% triethanolamine (pH 8.0) for 10 min. Between each step, the slides were washed twice in PBS (pH 7.5) for 5 min. The sections were then prehybridized in hybridization solution (0.3 M NaCl; 10 mM Tris-HCl, pH7.5; 1 mM EDTA; 1× Denhardt's; 5% dextran sulfate; 0.02% sodium dodecyl sulfate; 50% formamide; and 250 μg/ml salmon sperm DNA) at 42°C for 16 hrs and finally hybridized in 30 μl of the same solution containing 7.5 μg/mL DIG-labeled Lbx2 antisense or sense riboprobe at 42°C. On the next day, the slides were washed twice for 10 min at 42°C with 2 × SSC, 1 × SSC, 0.2 × SSC and 0.05 × SSC and incubated with a 1:1000 dilution of an alkaline phosphatase-conjugated anti-DIG antiserum (Roche Diagnostics, Laval, Canada) for 2 h at room temperature. Nitroblue tetrazolium chloride and 5-bromo-4-chloro-3-indolylphosphate p-toluidine (NBT/BCIP) were used as substrates for the alkaline phosphatase reaction. Sections were counterstained with 5% neutral red and mounted in Permount (Fisher Scientific, Montreal, Canada). The results presented were obtained with the 504 bp probe.

### Immunohistochemistry

Paraformaldehyde-fixed, paraffin-embedded testis sections were dewaxed in xylene, treated 30 min in 0.3% H_2_O_2 _(Sigma-Aldrich, Oakville, Canada)/methanol, rehydrated in graded alcohols (95%, 70%, and 50%) and treated for antigen retrieval. Sections were then blocked for 2 h with 10% horse serum and incubated overnight at 4°C with an Anti-Müllerian inhibitory substance antiserum (MIS, 1:100, Santa Cruz Biotechnology) in PBS containing 0.1% BSA. The next morning, the slides were washed in PBS and incubated 45 min with a biotinylated anti-goat antibody (1:1500, Vector Laboratories, Burlington, Canada). After washing in PBS, sections were submitted to an avidin-biotin complex (ABC) solution for 20 min at room temperature (Vectastain ABC Elite Kit, Vector Laboratories, Burlington, Canada). The signal was detected using a solution of 3-amino-9-ethylcarbazole (AEC, Sigma-Aldrich Canada, Oakville, Canada), 50 mM acetate buffer pH 5.2 (0.2 M sodium acetate; 0.2 M acetic acid) and 0.002% H_2_O_2_. Sections were then counterstained with Gill #1 hematoxylin and mounted in 15% glycerol and 0.1% sodium azide in PBS.

## Authors' contributions

VM collected tissues, performed all the experiments, and drafted the manuscript. DB collected tissues and participated in the writing of the manuscript. JJT conceived the study, coordinated and supervised the project, helped to draft the manuscript, and wrote the final version. All authors approved the final manuscript.

## Supplementary Material

Additional file 1 Strategy used to derive the degenerate PCR used to identify additional homeoproteins expressed in the male reproductive system. (A) Schematic representation of a homeoprotein with the homeodomain (HD) represented by a black box. DNA sequence alignment of the HD of 16 homeoproteins that have in common a lysine at position 50 of the homeodomain. Sequences corresponding to the two degenerate primers are shown by arrows. The HD is shown in black. (B) Sequences of the two degenerate primers. The expected size of the amplicon is 180 bp. Since the primers were located in different exons separated by an intron (not shown here), it was simple (based on predicted band sizes) to discriminate between genuine homeoproteins and amplification artifacts caused by any contaminating genomic DNA. Click here for file

Additional file 2 Expression of Lbx2 in Leydig cell lines by Northern blot. Total RNA from MA-10, mLTC-1, TM3 and R2C Leydig cell lines was extracted using the RNeasy Plus extraction kit (Qiagen, Mississauga, Ontario, Canada) and analyzed by Northern blot. Twenty μg of RNA were separated by agarose-formaldehyde gel electrophoresis and then transferred onto a nylon membrane (Hybond-N, GE Healthcare Life Sciences, Baie d'Urfé, Quebec, Canada). *Top panel*: membrane hybridization with a Lbx2 ^32^P-labeled cDNA probe was done using the QuikHyb Hybridization Solution as recommended by the manufacturer (Stratagene, La Jolla, CA, USA). The blot was washed under stringent conditions: 1 × SSC, 0.1% SDS for 30 min at 65°C and 0.1 × SSC, 0.1% SDS for 30 min at 65°C. Lover panel: to control for loading, the same membrane was stained with methylene blue. The position of 18S and 28S ribosomal RNA is indicated. Click here for file

## References

[B1] Desplan C, Theis J, O'Farrell PH (1985). The Drosophila developmental gene, engrailed, encodes a sequence-specific DNA binding activity. Nature.

[B2] Desplan C, Theis J, O'Farrell PH (1988). The sequence specificity of homeodomain-DNA interaction. Cell.

[B3] Hoey T, Levine M (1988). Divergent homeo box proteins recognize similar DNA sequences in Drosophila. Nature.

[B4] Bomgardner D, Hinton BT, Turner TT (2001). Hox transcription factors may play a role in regulating segmental function of the adult epididymis. J Androl.

[B5] Lindsey JS, Wilkinson MF (1996). Pem: a testosterone- and LH-regulated homeobox gene expressed in mouse Sertoli cells and epididymis. Dev Biol.

[B6] Viger RS, Silversides DW, Tremblay JJ (2005). New insights into the regulation of mammalian sex determination and male sex differentiation. Vitam Horm.

[B7] Hacker A, Capel B, Goodfellow P, Lovell-Badge R (1995). Expression of Sry, the mouse sex determining gene. Development.

[B8] Koopman P, Münsterberg A, Capel B, Vivian N, Lovell-Badge R (1990). Expression of a candidate sex-determining gene during mouse testis differentiation. Nature.

[B9] Teixeira J, Maheswaran S, Donahoe PK (2001). Mullerian inhibiting substance: an instructive developmental hormone with diagnostic and possible therapeutic applications. Endocr Rev.

[B10] Roy AK, Chatterjee B (1995). Androgen action. Crit Rev Eukaryot Gene Expr.

[B11] Nef S, Parada LF (1999). Cryptorchidism in mice mutant for Insl3. Nat Genet.

[B12] Zimmermann S, Steding G, Emmen JM, Brinkmann AO, Nayernia K, Holstein AF, Engel W, Adham IM (1999). Targeted disruption of the Insl3 gene causes bilateral cryptorchidism. Mol Endocrinol.

[B13] Kawamura K, Kumagai J, Sudo S, Chun SY, Pisarska M, Morita H, Toppari J, Fu P, Wade JD, Bathgate RA, Hsueh AJ (2004). Paracrine regulation of mammalian oocyte maturation and male germ cell survival. Proc Natl Acad Sci U S A.

[B14] Turner TT, Bomgardner D, Jacobs JP, Nguyen QA (2003). Association of segmentation of the epididymal interstitium with segmented tubule function in rats and mice. Reproduction.

[B15] Jones RC (1998). Evolution of the vertebrate epididymis. J Reprod Fertil Suppl.

[B16] Kirchhoff C (1999). Gene expression in the epididymis. Int Rev Cytol.

[B17] Jones RC (1999). To store or mature spermatozoa? The primary role of the epididymis. Int J Androl.

[B18] Miyamoto N, Yoshida M, Kuratani S, Matsuo I, Aizawa S (1997). Defects of urogenital development in mice lacking Emx2. Development.

[B19] Birk OS, Casiano DE, Wassif CA, Cogliati T, Zhao L, Zhao Y, Grinberg A, Huang S, Kreidberg JA, Parker KL, Porter FD, Westphal H (2000). The LIM homeobox gene Lhx9 is essential for mouse gonad formation. Nature.

[B20] Schnabel CA, Selleri L, Cleary ML (2003). Pbx1 is essential for adrenal development and urogenital differentiation. Genesis.

[B21] Kitamura K, Yanazawa M, Sugiyama N, Miura H, Iizuka-Kogo A, Kusaka M, Omichi K, Suzuki R, Kato-Fukui Y, Kamiirisa K, Matsuo M, Kamijo S, Kasahara M, Yoshioka H, Ogata T, Fukuda T, Kondo I, Kato M, Dobyns WB, Yokoyama M, Morohashi K (2002). Mutation of ARX causes abnormal development of forebrain and testes in mice and X-linked lissencephaly with abnormal genitalia in humans. Nat Genet.

[B22] Podlasek CA, Seo RM, Clemens JQ, Ma L, Maas RL, Bushman W (1999). Hoxa-10 deficient male mice exhibit abnormal development of the accessory sex organs. Dev Dyn.

[B23] Torres M, Gomez-Pardo E, Dressler GR, Gruss P (1995). Pax-2 controls multiple steps of urogenital development. Development.

[B24] Chen F, Liu KC, Epstein JA (1999). Lbx2, a novel murine homeobox gene related to the Drosophila ladybird genes is expressed in the developing urogenital system, eye and brain. Mech Dev.

[B25] Ohtoshi A, Nishijima I, Justice MJ, Behringer RR (2002). Dmbx1, a novel evolutionarily conserved paired-like homeobox gene expressed in the brain of mouse embryos. Mech Dev.

[B26] Wei K, Chen J, Akrami K, Sekhon R, Chen F (2007). Generation of mice deficient for Lbx2, a gene expressed in the urogenital system, nervous system, and Pax3 dependent tissues. Genesis.

[B27] Bouma GJ, Hart GT, Washburn LL, Recknagel AK, Eicher EM (2004). Using real time RT-PCR analysis to determine multiple gene expression patterns during XX and XY mouse fetal gonad development. Gene Expr Patterns.

[B28] Pellegrini M, Pantano S, Lucchini F, Fumi M, Forabosco A (1997). Emx2 developmental expression in the primordia of the reproductive and excretory systems. Anat Embryol (Berl).

[B29] Kanamoto T, Terada K, Yoshikawa H, Furukawa T (2006). Cloning and expression pattern of lbx3, a novel chick homeobox gene. Gene Expr Patterns.

[B30] Jagla K, Frasch M, Jagla T, Dretzen G, Bellard F, Bellard M (1997). ladybird, a new component of the cardiogenic pathway in Drosophila required for diversification of heart precursors. Development.

[B31] Jagla K, Jagla T, Heitzler P, Dretzen G, Bellard F, Bellard M (1997). ladybird, a tandem of homeobox genes that maintain late wingless expression in terminal and dorsal epidermis of the Drosophila embryo. Development.

[B32] Jagla T, Bellard F, Lutz Y, Dretzen G, Bellard M, Jagla K (1998). ladybird determines cell fate decisions during diversification of Drosophila somatic muscles. Development.

[B33] De GF, Jagla T, Daponte JP, Rickert C, Dastugue B, Urban J, Jagla K (2004). The ladybird homeobox genes are essential for the specification of a subpopulation of neural cells. Dev Biol.

[B34] Junion G, Bataille L, Jagla T, Da Ponte JP, Tapin R, Jagla K (2007). Genome-wide view of cell fate specification: ladybird acts at multiple levels during diversification of muscle and heart precursors. Genes Dev.

[B35] Schafer K, Neuhaus P, Kruse J, Braun T (2003). The homeobox gene Lbx1 specifies a subpopulation of cardiac neural crest necessary for normal heart development. Circ Res.

[B36] Muller T, Brohmann H, Pierani A, Heppenstall PA, Lewin GR, Jessell TM, Birchmeier C (2002). The homeodomain factor lbx1 distinguishes two major programs of neuronal differentiation in the dorsal spinal cord. Neuron.

[B37] Kruger M, Schafer K, Braun T (2002). The homeobox containing gene Lbx1 is required for correct dorsal-ventral patterning of the neural tube. J Neurochem.

[B38] Gross MK, Dottori M, Goulding M (2002). Lbx1 specifies somatosensory association interneurons in the dorsal spinal cord. Neuron.

[B39] Brohmann H, Jagla K, Birchmeier C (2000). The role of Lbx1 in migration of muscle precursor cells. Development.

[B40] Gross MK, Moran-Rivard L, Velasquez T, Nakatsu MN, Jagla K, Goulding M (2000). Lbx1 is required for muscle precursor migration along a lateral pathway into the limb. Development.

[B41] Schafer K, Braun T (1999). Early specification of limb muscle precursor cells by the homeobox gene Lbx1h. Nat Genet.

[B42] Dyche WJ (1979). A comparative study of the differentiation and involution of the Mullerian duct and Wolffian duct in the male and female fetal mouse. J Morphol.

[B43] Staack A, Donjacour AA, Brody J, Cunha GR, Carroll P (2003). Mouse urogenital development: a practical approach. Differentiation.

[B44] Sharpe RM, McKinnell C, Kivlin C, Fisher JS (2003). Proliferation and functional maturation of Sertoli cells, and their relevance to disorders of testis function in adulthood. Reproduction.

[B45] O'Shaughnessy PJ, Baker PJ, Johnston H (2006). The foetal Leydig cell - differentiation, function and regulation. Int J Androl.

[B46] Benton L, Shan LX, Hardy MP (1995). Differentiation of adult Leydig cells. J Steroid Biochem Mol Biol.

[B47] Mendis-Handagama SM, Ariyaratne HB (2001). Differentiation of the adult Leydig cell population in the postnatal testis. Biol Reprod.

[B48] Favier B, Dolle P (1997). Developmental functions of mammalian Hox genes. Mol Hum Reprod.

[B49] Bomgardner D, Hinton BT, Turner TT (2003). 5' hox genes and meis 1, a hox-DNA binding cofactor, are expressed in the adult mouse epididymis. Biol Reprod.

[B50] Drevet JR, Lareyre JJ, Schwaab V, Vernet P, Dufaure JP (1998). The PEA3 protein of the Ets oncogene family is a putative transcriptional modulator of the mouse epididymis-specific glutathione peroxidase gene gpx5. Mol Reprod Dev.

[B51] Maclean JA, Chen MA, Wayne CM, Bruce SR, Rao M, Meistrich ML, Macleod C, Wilkinson MF (2005). Rhox: a new homeobox gene cluster. Cell.

[B52] Benson GV, Lim H, Paria BC, Satokata I, Dey SK, Maas RL (1996). Mechanisms of reduced fertility in Hoxa-10 mutant mice: uterine homeosis and loss of maternal Hoxa-10 expression. Development.

[B53] Hsieh-Li HM, Witte DP, Weinstein M, Branford W, Li H, Small K, Potter SS (1995). Hoxa 11 structure, extensive antisense transcription, and function in male and female fertility. Development.

[B54] Jagla K, Dolle P, Mattei MG, Jagla T, Schuhbaur B, Dretzen G, Bellard F, Bellard M (1995). Mouse Lbx1 and human LBX1 define a novel mammalian homeobox gene family related to the Drosophila lady bird genes. Mech Dev.

[B55] Prince VE, Pickett FB (2002). Splitting pairs: the diverging fates of duplicated genes. Nat Rev Genet.

[B56] Lappin TR, Grier DG, Thompson A, Halliday HL (2006). HOX genes: seductive science, mysterious mechanisms. Ulster Med J.

[B57] Martienssen R, Irish V (1999). Copying out our ABCs: the role of gene redundancy in interpreting genetic hierarchies. Trends Genet.

[B58] Holland PW (2001). Beyond the Hox: how widespread is homeobox gene clustering?. J Anat.

[B59] Hatano M, Iitsuka Y, Yamamoto H, Dezawa M, Yusa S, Kohno Y, Tokuhisa T (1997). Ncx, a Hox11 related gene, is expressed in a variety of tissues derived from neural crest cells. Anat Embryol (Berl).

[B60] Ascoli M (1981). Characterization of several clonal lines of cultured Leydig tumor cells: gonadotropin receptors and steroidogenic responses. Endocrinology.

[B61] Ge RS, Dong Q, Sottas CM, Chen H, Zirkin BR, Hardy MP (2005). Gene Expression in Rat Leydig Cells During Development from the Progenitor to Adult Stage: A Cluster Analysis.. Biol Reprod.

[B62] Guigon CJ, Coudouel N, Mazaud-Guittot S, Forest MG, Magre S (2005). Follicular cells acquire sertoli cell characteristics after oocyte loss. Endocrinology.

