# Supplementary material for: Expression of the Ladybird-like homeobox 2 transcription factor in the developing mouse testis and epididymis
Source: BMC Dev Biol. 2008 Feb 27;8:22. doi: 10.1186/1471-213X-8-22 (PMC2277406; doi:10.1186/1471-213X-8-22)
Supplement: Additional file 1 — Strategy used to derive the degenerate PCR used to identify additional homeoproteins expressed in the male reproductive system. (A) Schematic representation of a homeoprotein with the homeodomain (HD) represented by a black box. DNA sequence alignment of the HD of 16 homeoproteins that have in common a lysine at position 50 of the homeodomain. Sequences corresponding to the two degenerate primers are shown by arrows. The HD is shown in black. (B) Sequences of the two degenerate primers. The expected size of the amplicon is 180 bp. Since the primers were located in different exons separated by an intron (not shown here), it was simple (based on predicted band sizes) to discriminate between genuine homeoproteins and amplification artifacts caused by any contaminating genomic DNA. [file 1471-213X-8-22-S1.pdf]

ADDITIONAL DATA FILE

EXPRESSION OF THE LADYBIRD-LIKE HOMEOBOX 2, LBX2, TRANSCRIPTION FACTOR IN THE DEVELOPING MOUSE TESTIS AND EPIDIDYMIS

Vanessa Moisan, Daniela Bomgardner and Jacques J. Tremblay

A

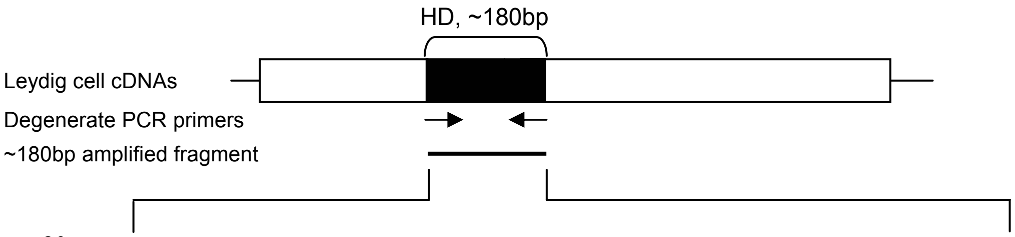


```
Munc30   CAGCAGCTGCAGGAGCTGGAAGCCACTTTCC .......... GTCCGGGTTTGGTTCAAGAATCGCCGGGCC
Alx4     T-C------G-A--------GAAAGTC---- .......... --G-A----------C----C--GA-----
Unc4     TG-------G----------GAAGG-G---A .......... -T--A---C------C-A------------
Dmbx1    --------TG---CC-----GAAA--C---- .......... --A-A---G-----T-----C---A-----
Prop1    GCA--------AC-------GT-AG-C--TG .......... A---A---C------C----C---A----T
Vsx1     --T--A--CG-A--A-----GAAGG-C--TG .......... A-A-A---C------C----C---A-----
Rax      T-C--A-----C--------GCG-G-C---G .......... ----A---A------C----C--A-----T
Prx1     AGC--A-------CCT----GCGTGTC--TG .......... --G-A---G-----TC----C--AA-A---
Prx2     AGC----------C-C----GCGTGTA--TG .......... ----AA--C------C----C-----T---
Pitx1    ------T----A--------G-----G---- .......... --G-----C-----------C--G--A---
Pitx2    ------------------------------- .......... ------------------------------
Pitx3    --------------------G-----C---- .......... --G-----G-----------C--G--C---
Crx      AGC------G----------G---CTG--TG .......... ----A---C--------------TC----G
Esx      ATC--A--------------G---TT----- .......... --G-A---------TC----C---A-T---
Otx1     TCA------G-C-T---C--G--GCTG---G .......... ----A---------------C-----C---
Otx2     GCA-----CG-C-TT-----G-T-TG---G- .......... --G-A---A-----T--------AA-A--T
```

Degenerate Primer 1 → ← Degenerate Primer 2

B

Degenerate primer 1	*Forward*	5'-GATCTAGASCARCTGSAGGMGCTGGAG-3'
Degenerate primer 2	*Reverse*	5'-GCGGTACCGCBCKSCGGTTCTKRAACCA-3'
