# Supplementary material for: Expression of the Ladybird-like homeobox 2 transcription factor in the developing mouse testis and epididymis
Source: BMC Dev Biol. 2008 Feb 27;8:22. doi: 10.1186/1471-213X-8-22 (PMC2277406; doi:10.1186/1471-213X-8-22)

ADDITIONAL DATA FILE

EXPRESSION OF THE LADYBIRD-LIKE HOMEOBOX 2, LBX2, TRANSCRIPTION FACTOR IN THE DEVELOPING MOUSE TESTIS AND EPIDIDYMIS

Vanessa Moisan, Daniela Bomgardner and Jacques J. Tremblay

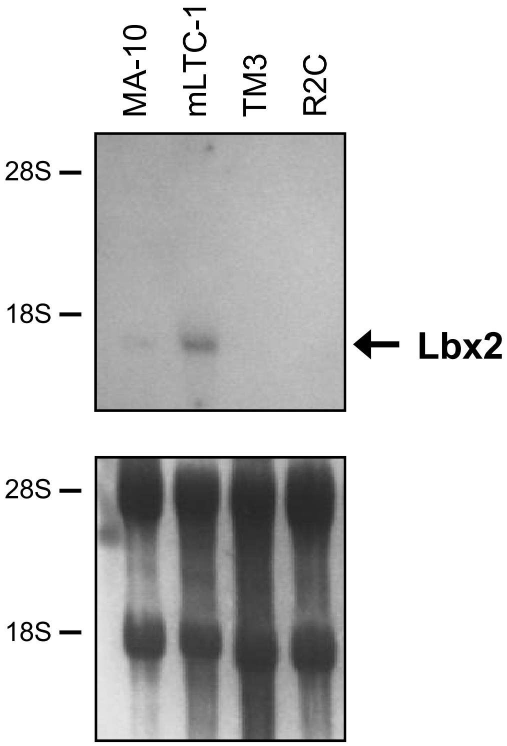

Supplement: Additional file 2 — Expression of Lbx2 in Leydig cell lines by Northern blot. Total RNA from MA-10, mLTC-1, TM3 and R2C Leydig cell lines was extracted using the RNeasy Plus extraction kit (Qiagen, Mississauga, Ontario, Canada) and analyzed by Northern blot. Twenty μg of RNA were separated by agarose-formaldehyde gel electrophoresis and then transferred onto a nylon membrane (Hybond-N, GE Healthcare Life Sciences, Baie d'Urfé, Quebec, Canada). Top panel: membrane hybridization with a Lbx2 32P-labeled cDNA probe was done using the QuikHyb Hybridization Solution as recommended by the manufacturer (Stratagene, La Jolla, CA, USA). The blot was washed under stringent conditions: 1 × SSC, 0.1% SDS for 30 min at 65°C and 0.1 × SSC, 0.1% SDS for 30 min at 65°C. Lover panel: to control for loading, the same membrane was stained with methylene blue. The position of 18S and 28S ribosomal RNA is indicated. [file 1471-213X-8-22-S2.pdf]
